# Editorial: Circadian rhythm in metabolism and endocrinology

**DOI:** 10.3389/fendo.2023.1263823

**Published:** 2023-08-02

**Authors:** Maria Rodriguez-Fernandez

**Affiliations:** ^1^ Institute for Biological and Medical Engineering, Schools of Engineering, Medicine and Biological Sciences, Pontificia Universidad Catolica de Chile, Santiago, Chile; ^2^ Millenium Institute for Intelligent Healthcare Engineering iHealth, Santiago, Chile

**Keywords:** circadian rhythm, metabolism, endocrinology, sleep patterns, chronopharmacology

Circadian rhythms play a fundamental role in our daily lives, orchestrating a wide range of physiological processes, including energy metabolism, immune function, cellular rejuvenation, and the intricate interplay with the gut microbiota. This Research Topic delves into the profound influence of circadian rhythms on various aspects of metabolism and endocrinology and explores the intricate connections between circadian physiology and metabolic disorders. By elucidating these relationships, this Research Topic aims to provide insights into the development of novel therapeutic strategies for the management of metabolic and endocrine diseases.

At the population level, Li et al. conducted a study revealing an independent association between rest-activity rhythm and obesity phenotypes. Their findings highlight the impact of aberrant rest-activity rhythm on anthropometric and imaging measures of general and abdominal obesity.

Examining the intergenerational impact of circadian disruptions, Huang et al. explore the effects of mistimed feeding on circadian reprogramming and the unfolded protein response. Their study demonstrates the cumulative impact of mistimed food intake over multiple generations, resulting in altered diurnal rhythmic transcriptomes and impaired endoplasmic reticulum stress response.

Sun et al. conducted a bidirectional Mendelian randomization study providing genetic evidence that supports a bidirectional causal relationship between sleep traits and nonalcoholic fatty liver disease (NAFLD). Their findings emphasize the importance of considering sleep disturbances in managing NAFLD.

Shifting the focus to pharmacology, Cortés-Ríos et al. used mathematical modeling to investigate the dosing-time-dependent antihypertensive effect of valsartan and aspirin. Their study sheds light on the optimal dosing time for these medications, underscoring the importance of chronopharmacology in hypertension management.

Expanding the scope to sleep patterns, Wild et al. conducted a case-control study evaluating sleep patterns in patients treated for non-secreting intra- and parasellar tumors. Their findings revealed altered sleep patterns in these patients compared to healthy controls, highlighting the need for clinicians to address sleep disturbances in this population.

Moving to a more specific endocrine system, Bao et al. performed a prospective clinical trial investigating the effect of melatonin on the quality of repeated-poor and frozen-thawed embryos in humans. Their study demonstrates that melatonin supplementation in the culture medium improves the rate of high-quality embryos, offering a potential rescue strategy for *in vitro* fertilization (IVF) failures.

Exploring the potential therapeutic application of melatonin, Yin et al. reviewed the use of melatonin for premenstrual syndrome (PMS). They discuss the role of melatonin in modulating sleep disturbance, mood changes, and cognitive impairment associated with PMS, suggesting its potential as a safe and effective treatment option.

Lastly, Watanabe et al. explored the insulin-independent action of nocturnal melatonin in increasing glucose uptake in the goldfish brain. Their study highlights the role of melatonin in regulating glucose homeostasis and its potential as a circadian regulator of glucose dynamics.

The articles included in this Research Topic provide valuable insights into the intricate relationship between circadian rhythm, metabolism, and endocrinology. From the impact of mistimed feeding on circadian reprogramming to the bidirectional relationship between sleep traits and NAFLD, the findings from Li et al., Huang et al., Sun et al., Cortés-Ríos et al., Wild et al., Bao et al., Yin et al., and Watanabe et al. emphasize the importance of circadian regulation and sleep patterns in maintaining metabolic homeostasis and offer potential avenues for therapeutic interventions in metabolic and endocrine disorders. The comprehensive understanding of the ties between circadian rhythms and metabolism presented in this Research Topic will undoubtedly pave the way for innovative approaches in the management of these conditions.

## Author contributions

MR-F: Writing – original draft, Writing – review & editing

